# Involvement of Carbohydrate, Protein and Phenylanine Ammonia Lyase in Up-Regulation of Secondary Metabolites in *Labisia pumila* under Various CO_2_ and N_2_ Levels

**DOI:** 10.3390/molecules16054172

**Published:** 2011-05-20

**Authors:** Mohd Hafiz Ibrahim, Hawa Z.E. Jaafar

**Affiliations:** Department of Crop Science, Faculty of Agriculture, University Putra Malaysia, 43400 Serdang, Selangor, Malaysia; Email: mhafizphd@yahoo.com (M.H.I.)

**Keywords:** CO_2_ enrichment, total phenolics, total flavonoids, carbon-to-nitrogen ratio, total non structurable carbohydrates, total soluble sugar and starch profiling, Kacip Fatimah

## Abstract

A split plot factorial 2 × 3 experiment was designed to examine and characterize the relationships among secondary metabolites (total phenolics, TP; total flavonoids, TF), carbohydrate content, C/N ratio, protein synthesis and L–phenylalanine ammonia lyase (PAL; EC 4.3.1.5) activity in the Malaysian medicinal herb *Labisia pumila* (Blume) Fern-Vill. under different CO_2_ concentrations (400 = ambient and 1,200 µmol mol^−1^ CO_2_) and three levels of nitrogen fertilization (0, 90 and 270 kg N ha^−1^) for 15 weeks. The interaction between CO_2 _and nitrogen levels imposed a significant impact on plant secondary metabolite production, protein, PAL activity and fructose levels. Highest TP and TF were recorded under 1,200 µmol mol^−1^ CO_2_ when N fertilizer was not applied; lowest values were obtained at 400 µmol mol^−1^ CO_2_ fertilized with the highest N level. Concurrently, fructose contents increased tremendously. Increase in fructose content might also enhance erythose-4-phosphate production (substrate for lignin and phenolic compounds), which shares a common precursor transdalolase in the pentose phosphate pathway. PAL activity was noted to be highest under 1,200 µmol mol^−1^ CO_2_ + 0 kg N ha^−1 ^coinciding with subsequent recording of the lowest protein content. The results implied that the increase in plant secondary metabolites production under the tested conditions might be due to diversion of phenylalanine for protein synthesis to production of secondary metabolites. It was also found that the sucrose to starch ratio was also high under high levels of nitrogen fertilization, indicating an enhanced sucrose phosphate synthase activity (SPS; EC 2.4.1.14) under such condition.

## Abbreviations

TP: total phenolics
TF: total flavonoid
PAL: Phenyll alanine lyase
N: nitrogen
C/N: Carbon to nitrogen ratio
SPS: Sucrose phosphate synthase
ROS: Reactive oxygen species
CNB: carbon nutrient balance
GDB: Growth differentiation balance
TNC: Total non structurable carbohydrate
CBSM: Carbon based secondary metabolites

## 1. Introduction

*Labisia pumila* (Blume) Fern-Vill., locally known as Kacip Fatimah, Selusoh Fatimah or Akar Kacip Fatimah, is a sub-herbaceous plant with creeping stems from the family Myrsinaceae that is found widespread in Indochina and throughout the Malaysian forest [1]. It is a shade loving plant and grows well under thinned jungle with 70% to 90% shade. In Peninsular Malaysia, it is widely distributed throughout a few states like Perak, Pahang, Selangor and Negeri Sembilan [2]. Traditionally *L. pumila* has been used by Malay women to induce and facilitate childbirth as well as in post-partum medicine [3]. Stone [4] has categorized three varieties of this herb in Malaysia, namely *L. pumila* (Blume) Fern-Vill var. *alata*, *L. pumila* (Blume) Fern-Vill var. *pumila* and *L. pumila* (Blume) Fern-Vill var. *lanceolata*. Each of the varieties has different uses. The most universally utilized varieties by the traditional healers are the first two, *L. pumila* var. *alata* and *L. pumila* var. *pumila.* The other uses include treatments for dysentery, dysmenorrhea, flatulence and gonorrhea [5]. *L. pumila* is widely and commercially available as a health supplement. 

Previous studies have indicated that the bioactive compounds of *L. pumila* consisted of resorcinols, flavonoids and phenolic acids [1,6]. These compounds are all phenolics, and possess a wide range of structures that contribute to the organoleptic and nutritional qualities of fruits and vegetables [7]. These phytochemicals are often referred as antioxidants on account of their ability to protect the plant against damages caused by reactive oxygen species (ROS), which are involved in various diseases such as cancer and atheroscelorosis. The uptake of high levels of antioxidant supplements can reduce the risk of these diseases. Phenolic compounds have been implicated as natural antioxidants that may reduce oxidative damage to the human body [8]. Recent work by Norhaiza *et al.* [9] has shown that *L. pumila* have high antioxidant properties attributable to the presence these bioactive compounds.

Environmental factors such as nutrient supply, temperature, light conditions or atmospheric CO_2_ concentrations can influence the levels of carbon based secondary metabolites in plant tissues, and plant partitioning of carbohydrates and energy [10]. As it is generally known that secondary metabolism is linked to primary metabolism by the rates at which substrates are diverted from primary pathways and funneled into the secondary biosynthetic routes, several environmental factors affecting growth, photosynthesis and other parts of primary metabolism will also affect secondary metabolism. One of these factors is CO_2_, and the increases in atmospheric CO_2_ due to climate change have a direct impact on plant secondary metabolites. Lately, it has been found that the enrichment of *L*. *pumila* with high levels of CO_2_ increased the secondary metabolite production (phenolics and flavonoids) of this plant [11]. A similar result was also observed in ginger (*Zingiber officinale*) [12].

In most studies, it is reported that concentrations of some carbon based secondary metabolites (CBSM) such as phenolics usually increase under elevated CO_2_[13]. Levels of CBSM are partly determined by environmental conditions [14]. Resource allocation hypotheses such as the carbon-nutrient balance (CNB) [15] and growth differentiation balance (GDB) [16] have been proposed by researchers to predict the effects of environmental factors. They assume that changes in carbon source-sink relationship, as a consequence of reducing nutrient availability or modifying carbon availability, determines variations in the relative partitioning of carbon to growth, carbon based secondary metabolites (CBSM) and total non structural carbohydrates (TNC). The predictions of these hypotheses can be tested by exposing plants to increasing CO_2_ levels. The consequent rise in carbon-nutrient availability limits the growth more than photosynthesis, which results in accumulation of TNC and a decrease in nitrogen [17].

When protein synthesis is restricted under high carbon-nitrogen availability ratio, the consequent lower demand of amino acids could determine the stimulation of phenolic compounds synthesis [18]. Two hypotheses have been proposed to explain the increases of CBSM under these conditions. Bass [19] considered that TNC in excess of those required for protein synthesis as the main factor affecting the increase of CBSM under elevated CO_2_. On the other hand, Jones and Hartley [14] and Lambers [20] proposed the involvement of the amino acid phenylalanine as a common precursor for proteins and phenolics. Competion between proteins and phenolics for limiting phenylalanine would result in a trade-off between protein and phenolics synthesis rates, thus reversing the relationship between protein and phenolics allocation. These two hypotheses consider the increase of CBSM under elevated CO_2_ as a result of a metabolic excess of carbon with no physiological cost on growth. A negative relationship between CBSM and growth are expected because increases in growth along a gradient of increasing nutrient availability will result in less carbon being available for CBSM production [15].

Many studies have investigated the effects of elevated CO_2_ on plant primary metabolism but relatively few studies have investigated the response of plant CBSM to increasing CO_2_ and its interaction with nitrogen availability. The objective of this study was to examine the effects of different CO_2_ and nitrogen levels on plant total carbon, leaf nitrogen, C/N ratio, protein synthesis, PAL activity, primary (soluble sugar, starch) and secondary metabolite (flavonoids and phenolics) syntheses in the medicinal herb *L. pumila*. The relationships among protein synthesis, PAL activity, carbohydrate, and total phenolics and flavonoids of plants exposed to combined CO_2_ enrichment and nitrogen levels were also determined.

## 2. Results and Discussion

### 2.1. Glucose and Sucrose

The glucose and sucrose levels were influenced by elevated CO_2_ levels (P ≤ 0.05; Table 1). The levels were found to be higher under 1,200 µmol mol^−1^ CO_2_ than 400 µmol mol^−1^ CO_2_ (ambient conditions). The glucose and sucrose contents were 28% and 49% higher in 1,200 µmol mol^−1^ CO_2_ than under ambient CO_2_ concentration, respectively. The increase in sucrose and glucose under the enriched condition has also been observed in other plants such as tomatoes, potatoes, orchids and sugarcane [21,22,23,24]. These increases might be due to an increase in hexose phosphate production under elevated CO_2_. Hexose phosphate is a precursor for sucrose synthesis so as hexose phosphate levels increased the production of sucrose and glucose was enhanced too [25]. 

**Table 1:** Effects of elevated CO_2_ on glucose and sucrose concentration of *L. pumila.*

CO_2_ levels (µmol mol^−1^)	Glucose (mg g^−1^ dry weight)	Sucrose (mg g^−1^ dry weight)
400	11.89 ± 0.32 b	36.32 ± 1.00 b
1200	15.27 ± 0.66 a	54.34 ± 0.90 a

All analyses are mean ± standard error of mean (SEM). N = 15. Means not sharing a common letter were significantly different at P ≤ 0.05.

### 2.2. Fructose

**Figure 1:**
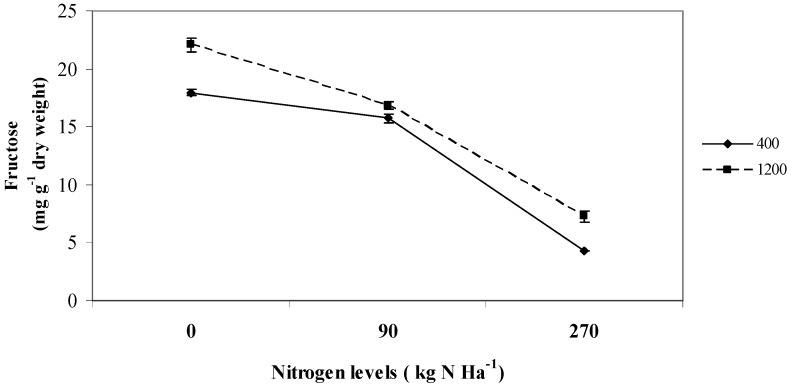
Interaction effects between CO_2_ (µmol mol^−1^) and nitrogen levels on fructose content of *L. pumila*. N = 12. Bars represent standard error of differences between means (SEM).

Fructose levels in *L. pumila* were influenced by the interaction effects between CO_2_ and nitrogen (P ≤ 0.01; Figure 1). The highest fructose level was produced under 0 kg N ha^−1^ and was higher at 1,200 µmol mol^−1^ CO_2_ (22.07 mg g^−1^ dry weight) than 400 µmol mol^-1^ CO_2_ (17.95 mg g^−1^ dry weight). 

As nitrogen fertilization was increased to 270 kg N ha^−1^ the fructose content decreased, especially under ambient CO_2_ (4.32 mg g^−1^ dry weight) compared to the plant at 1,200 µmol mol^−1^ CO_2_ that recorded a dry weight of 7.26 mg g^−1^. A similar observation was highlighted by Sun *et al.* [26] in *Arabidopsis* sp. where the interaction between high CO_2_ and low nitrogen levels increased the production of fructose; and a decrease in fructose content at ambient CO_2_ and high nitrogen fertilization was noted. However, similar work by Pilar *et al.* [27] on wheat did not find any significant interaction effects between CO_2_ and nitrogen in influencing the fructose concentration in wheat leaves. 

**Table 2:** Correlations among the measured parameters in the experiments.

		1	2	3	4	5	6	7	8	9	10	11	12
**1**	**Glucose**	1.000											
**2**	**Sucrose**	0.316	1.000										
**3**	**Fructose**	0.121	0.352	1.000									
**4**	**Starch**	0.175	0.39	0.942 *	1.000								
**5**	**TNC ^1^**	0.305	0.602 *	0.927 *	0.963 *	1.000							
**6**	**CN ^2^**	−0.008	0.136	0.799 *	0.809 *	0.730 *	1.000						
**7**	**Phenolics**	0.207	0.457	0.948 *	0.929 *	0.939 *	0.788 *	1.000					
**8**	**Flavonoids**	0.166	0.379	0.942 *	0.947 *	0.926 *	0.852 *	0.955 *	1.000				
**9**	**PAL ^3^**	0.048	0.241	0.740 *	0.814 *	0.751 *	0.788 *	0.744 **	0.842 *	1.000			
**10**	**Nitrogen**	0.102	−0.049	−0.853 *	−0.832 *	−0.728 *	−0.929 *	−0.785 *	−0.841 *	−0.777 *	1.000		
**11**	**Protein**	−0.023	−0.139	−0.798 **	−0.841 *	−0.754 *	−0.862 *	−0.714 *	−0.812 *	−0.784 *	0.899 *	1.000	
**12**	**Suc/ starch ^4^**	0.128	0.442	−0.629 *	−0.645 *	−0.434	−0.671 *	−0.519 *	−0.600 *	−0.589 *	0.780 *	0.709 *	1.000

* and ** = significant at 5% and 1% respectively. 1 = Total non structurable carbohydrate; 2 = carbon to nitrogen ratio; 3 = Phenyl alanine lyase activity and 4 = Sucrose / Starch ratio.

It was found that fructose had a strong significant positive correlation with starch (R^2^ = 0.942; P ≤ 0.05), as presented in the correlation Table 2. This showed that as starch levels increased in the leaves, fructose production might also be upregulated. The same positive correlation between starch and fructose was observed by Rose *et al.* [28] in quinoa seedlings, which implied that the increase in fructose might be due to increase in starch production in the leaves.

### 2.3. Starch and Total Non Structural Carbohydrates (TNC)

The starch and total non structural carbohydrate were influenced by the nitrogen levels applied to *Labisia pumila* seedlings (P ≤ 0.05). From Table 3 it is observed that as the nitrogen fertilization increased, the starch and total non structural carbohydrate decreased significantly from 0 kg N ha^−1^ > 90 kg N ha^−1^ > 270 kg N ha^−1^. 

**Table 3:** The effects of nitrogen levels on starch and total non structural carbohydrate.

Nitrogen levels (Kg N ha^−1^)	Starch (mg g^−1^ glucose dry weight)	Total non structural carbohydrate (mg g^−1^ dry weight)
0	116.29 ± 3.12 a	192.94 ± 9.12 a
90	102.58 ± 3.49 b	179.27 ± 8.52 b
270	76.16 ± 2.73 c	141.65 ± 7.75 c

All analyses are mean ± standard error of mean (SEM), N = 15. Means not sharing a common letter were significantly different at P ≤ 0.05.

**Figure 2:**
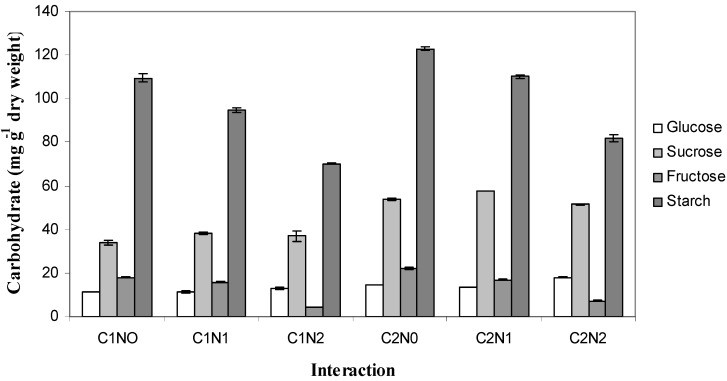
Interaction effects between CO_2_ and Nitrogen levels on carbohydrate partitioning in *L. pumila*. N = 6. Treatments used were C1N0 = 400 µmol mol^−1^ CO_2_ + 0 kg N ha^−1^; C1N1 = 400 µmol mol^−1^ CO_2_ + 90 kg N ha^−1^; C1N2 = 400 µmol mol^−1^ CO_2_ + 270 kg N Ha^−1^; C2N0 = 1,200 µmol mol^−1^ CO_2_ + 0 kg N Ha^−1^; C2N1 = 1,200 µmol mol^−1^ CO_2_ + 90 kg N Ha^−1^; C2N2 = 1,200 µmol mol^−1^ CO_2_ + 270 kg N Ha^−1^. Bars represent standard error of differences between means (SEM).

At end of 15 weeks after start of treatment (WAT), the starch content in 0 kg N ha^−1^ was 116.29 mg g^−1^ dry weight, followed by 90 kg N ha^−1^ (102.58 mg g^−1^ dry weight) and 270 kg N ha^−1^ (76.16 mg g^−1^ dry weight). Under nitrogen treatments, starch and TNC were highest at 0 kg N ha^−1^ and decreased with increasing N fertilization until reaching the lowest value at 270 kg N ha^−1^. The TNC decreased by 7% and 27%, respectively, in 90 and 270 kg N ha^−1^ compared to the ones under 0 kg N ha^−1^. With increased CO_2_ concentration from 400 to 1,200 µmol mol^−1^, this resulted in the highest values in starch and TNC. However, the effect of nitrogen was found to be stronger than CO_2_ enrichment in modifying the starch and TNC levels, resulting in higher values under low nitrogen levels than elevated CO_2_ ones Similar findings were also obtained by other workers [29,30,31] where they found increased levels of starch and TNC under high CO_2_ concentration and low nitrogen levels; there were no interaction effects observed between CO_2_ and nitrogen levels. In the present study, the increase in starch content might influence the increase in TNC. From Figure 2, starch content showed the highest carbohydrate values in all interaction treatments. This signifies the role of starch in influencing TNC effects. Furthermore, from the correlation table, starch and TNC showed a strong positive correlation (R^2^ = 0.963; P ≤ 0.05), which suggested that the increase in TNC had contributed, particularly, to the increase of the starch content of the leaves. The increase in starch and total soluble sugar (TSS) might be due to a decline in organic phosphate (P_i_) and increase in phosphoglyceric acid [32].

### 2.4. Carbohydrate Partitioning

The carbohydrate partitioning in the leaves of *L. pumila* is demonstrated in Figure 2. In all the combination treatments, starch was found to be the highest carbohydrate component accumulated, followed by sucrose. Plants that were fertilized with 0 and 90 kg N ha^−1^ displayed fructose as the highest carbohydrate after sucrose, and that for 270 kg N ha^−1^, glucose was the third highest carbohydrate after sucrose, whilst fructose concentration under ambient CO_2_ and 270 kg N ha^−1^ was the lowest. It was also observed that starch content decreased with increasing nitrogen fertilization under elevated and ambient CO_2_ concentration.

### 2.5. Leaf C to N Ratio and Nitrogen

The C/N ratio and nitrogen levels were influenced by the nitrogen fertilizer applied to the seedlings (Table 4). At the end of 15 WAT, the C/N ratio of 0 kg N ha^−1^ treatment had registered a value of 24.39, followed by 90 kg N ha^−1^ and 270 kg N ha^−1^ with C/N values of 16.473 and 9.402, respectively. The high plant C/N values might be contributed to by the reduction in nitrogen content rather than increases in total carbon. Similar findings were reported by Zhao *et al.* [33], Aguera *et al.* [34] and Coviella *et al.* [35]. In the present study, the increase in plant C/N ratio might be an indicator of an increase in plant secondary metabolites. This was shown from the correlation Table 2 where total phenolics and flavonoids had significant positive values of R^2^ = 0.788 and 0.852 (P ≤ 0.05), respectively. Winger *et al.* [36] indicated that increases in the C/N ratio in plants was an indication of increases in the synthesis of plant secondary metabolites, especially phenolics and flavonoids. The increase in plant C/N ratio signifies that an increase in the production of TNC that might stimulate the production of plant secondary metabolites [37,38]. 

**Table 4:** Effects of nitrogen levels on C/N ratio and leaf nitrogen content in *L. pumila.*

Nitrogen levels (Kg N ha^−1^)	C/N	Leaf nitrogen content (%)
0	24.39 ± 1.47 a	1.74 ± 0.08 c
90	16.47 ± 0.68 b	2.61 ± 0.11 b
270	9.40 ± 0.27 c	4.53 ± 0.07 a

All analyses are mean ± standard error of mean (SEM), N = 15. Means not sharing a common single letter were significantly different at P ≤ 0.05.

In the present study, this statement was proven when the C/N ratio displayed a significant positive relationship with TNC (R^2^ = 0.730; P ≤ 0.05). However, the leaf nitrogen registered a negative relationship with C/N ratio when nitrogen levels increased with increasing N fertilization. Although there was no nitrogen fertilization at 0 kg N ha^−1^, the leaf nitrogen content of the seedlings had indicated sufficient nitrogen to support growth [39]. From the correlation Table 2, it was shown that leaf nitrogen content has a significant negative relationship with the production of plant secondary metabolites, phenolics (R^2^ = −0.785; P ≤ 0.05) and flavonoids (R^2^ = −0.841; P ≤ 0.05). These data indicated that the production of plant secondary metabolites might be upregulated when the plants were under limitation of nitrogen [40].

### 2.6. Total Phenolics and Flavonoids

**Figure 3:**
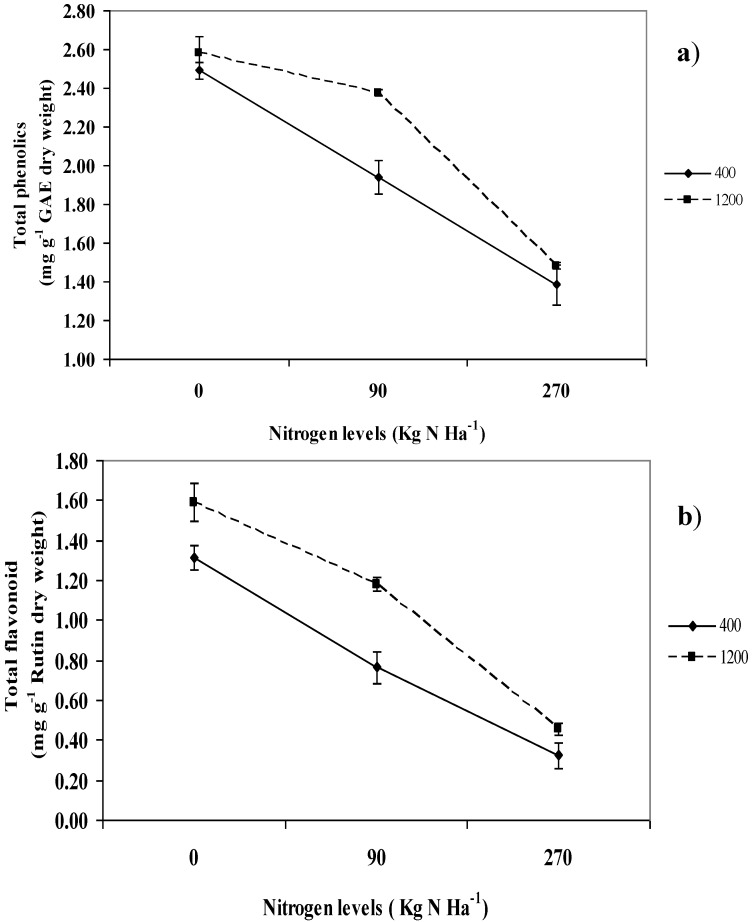
Interaction effects between CO_2_ and nitrogen levels on total phenolics (**a**) and total flavonoids (**b**) content of *L. pumila*. N = 12. Bars represent standard error of differences between means (SEM).

Total phenolics and flavonoids were influenced by interaction between CO_2_ and nitrogen levels (P ≤ 0.05; Figure 3). It was observed that the production of plant secondary metabolites was highest under 0 kg N ha^−1^ under elevated CO_2_ (1,200 µmol mol^−1^), compared to the ambient CO_2_ levels (400 µmol mol^−1^). The lowest production of total phenols and flavonoids was observed at 270 kg N ha^−1^ under ambient CO_2_ conditions compared to those plants enriched with 1,200 µmol mol^−1^ CO_2_. 

Total phenolics and flavonoids were higher under elevated CO_2_ than ambient CO_2_ combined with 0, 90 and 270 kg ha^−1^ nitrogen. Correlation analysis (Table 2) revealed that the total phenolics and flavonoids had positive significant relationships with fructose (0.948 and 0.942; P ≤ 0.05), suggesting that that the increase in production of plant secondary metabolites might be due to an increase in fructose content. Furthermore, the increase in fructose was contributed by interaction effects between CO_2_ and nitrogen level, similar to those of total phenolics and flavonoids. The contribution of fructose in increasing plant secondary metabolites was reported once, and it is well known that an increase in soluble sugar is involved in the upregulation of the production of secondary metabolites in plants [12,40,41]. The increase in production of secondary metabolites by the increase in fructose content have been observed by Mirna *et al.* [42] in quinoa seedlings where the increase in total phenolics and flavonoids of the seedlings under UV-B radiation was significantly related to an increase in fructose content in quinoa leaves. The positive relationship between fructose and secondary metabolites might be due to enhanced activity of penthose phosphate pathway to supply high levels of erythose-4-phoshate, which is used as a substrate for the synthesis of lignin and secondary metabolite compounds in the shikimate acid pathway [43]. The increase in the production of erythose-4-phoshate implies a high production of fructose because both these compounds are synthesized in the same reaction catalyzed by transaldolase in the penthose phosphate pathway [42]. The current result suggest that under high CO_2_ and low nitrogen levels, the production of secondary metabolites was upregulated, and this might have been attributed to increase in penthose phosphate pathway due to increased erythose-4-phoshate synthesis, which is the precursor to plant secondary metabolites.

### 2.7. Phenylalanine-lyase; PAL Activity

**Figure 4:**
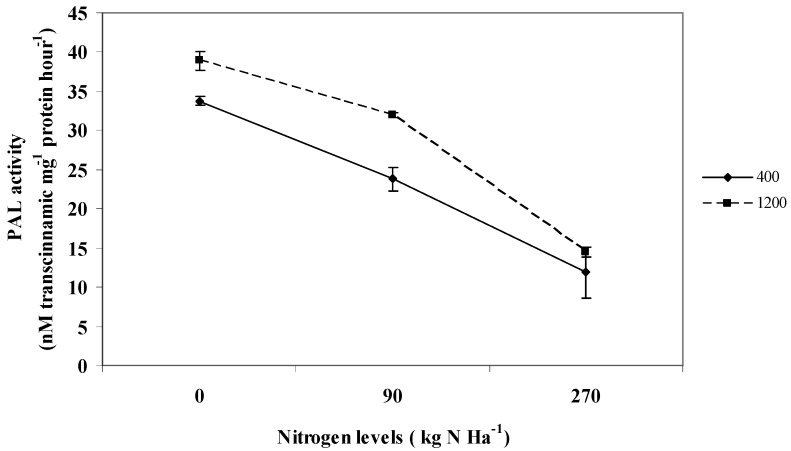
Interaction effects between CO_2_ and nitrogen levels on PAL activity of *L. pumila*. N = 12. Bars represent standard error of differences between means (SEM).

The PAL activity was influenced by interaction effects between CO_2_ and nitrogen levels (P ≤ 0.05; Figure 4). The PAL activity was found to be higher under 0 kg N ha^−1^ when the CO_2_ level was the highest (38.9 nM transcinnamic mg^−1^ protein hour^−1^) than under ambient CO_2_ (33.75 nM transcinnamic mg^−1^ protein hour^−1^). 

As the levels of nitrogen increased from 0 kg to 90 kg and 270 kg N ha^−1^ the activity of PAL decreased, registering the lowest PAL values at ambient CO_2_ under 270 kg N ha^−1^ (11.8 nM transcinnamic mg^−1^ protein hour^−1^). The increase in production of secondary metabolites in the present work could be due to increase in PAL activities under high CO_2_ combined with low nitrogen levels. It was also found that interaction effects between CO_2_ and nitrogen levels influenced the PAL activity. Correlation analysis showed that PAL had a significant positive relationship with total phenolics and flavonoids (R^2^ = 0.744; R^2^ = 0.842; P ≤ 0.05), which might indicate an upregulation of plant secondary metabolite production with increased PAL activity. This is basically due to the fact that PAL is a precursor to total phenolics and flavonoids biosynthesis [14]. The increase in PAL activity under high CO_2_ combined with low nitrogen level was also observed by Matros *et al.* [44] and Hartley *et al.* [45] in tobacco and *Spergula avensis*. These results suggest that upregulation of production of plant secondary metabolites in *L. pumila* under high CO_2_ and low nitrogen might be due to an increase in PAL activity.

### 2.8. Soluble Protein

**Figure 5:**
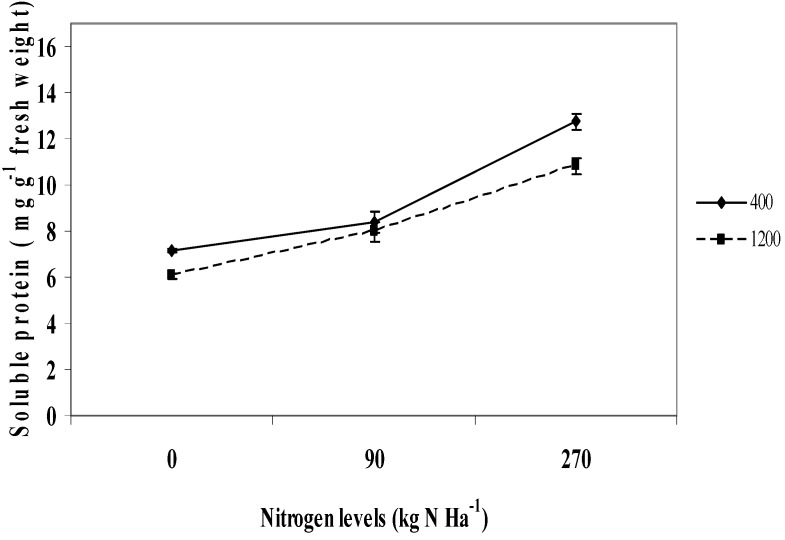
Interaction effects between CO_2_ and nitrogen levels on soluble protein content of *L. pumila*. N = 12. Bars represent standard error of differences between means (SEM).

Soluble protein of *L. pumila* was influenced by the interaction between CO_2_ and nitrogen levels (P ≤ 0.01). As the levels of nitrogen increased from 0 to 270 kg N ha^−1^, the soluble protein also increased, but the increase in soluble protein was highest under ambient CO_2_ compared to 1,200 µmol mol^−1^ CO_2_ level (Figure 5). The highest protein content was obtained in 400 µmol mol^−1^ CO_2_ when seedlings were fertilized with 270 kg ha^−1^ N (12.759 mg g^−1^ dry weight), and the lowest in 1,200 µmol mol^−1^ CO_2_ without any N fertilizer (6.06 mg g^−1^ dry weight). Similar results to those of the present study was observed by Erbs *et al.* [28] and Gleadow *et al.* [46] in wheat and cassava, where the highest protein accumulation was observed under ambient CO_2_ and high nitrogen fertilization. Protein content was also found to have a negative relationship with total phenols and flavonoids (R^2^ = −0.714; R^2^ = −0.812; P ≤ 0.05), which indicates the occurrence of an upregulation of plant secondary metabolites when protein content was reduced [47]. In the present study, the decreased protein production under high CO_2_ and low nitrogen levels might decrease the use of PAL for protein synthesis which is necessary for the biosynthesis of plant secondary metabolites [48]. This explains why increased in secondary metabolites might be upregulated under the current conditions. 

### 2.9. Sucrose to Starch Ratio

**Figure 6:**
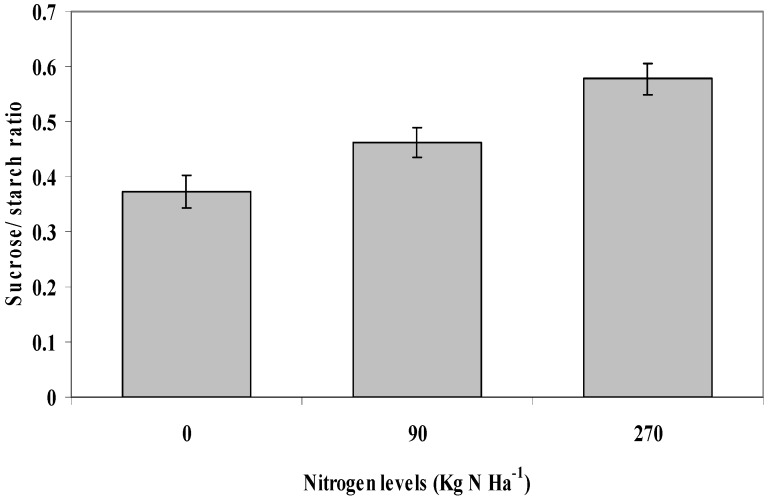
The effects of nitrogen levels on sucrose/starch ratio of *L. pumila*. N = 12. Bars represent standard error of differences between means (SEM).

The sucrose/starch ratio was only influenced by the nitrogen levels applied to the *L. pumila* seedlings (P ≤ 0.05; Figure 6). The sucrose/starch ratio in 0 kg N ha^−1^ was 0.373, followed by 90 kg N ha^−1^ (0.462) and 270 kg N ha^−1^ (0.577). 

These results showed that as the nitrogen levels increased to reach 270 kg N ha^−1^ the sucrose/starch ratio also increased. The sucrose/starch ratios in 90 and 270 kg N ha^−1^ were 23% and 57%, respectively, higher than at 0 kg N ha^−1^. Sucrose/starch ratio is an indication of sucrose phosphate synthase activity (SPS) in plants [49]. In the present study, SPS activity was shown to be highest in 270 kg N ha^−1^, followed by 90 kg N ha^−1^ and the least at 0 Kg N ha^−1^. As indicated in the present work, SPS activity was highest when the level of nitrogen fertilization was high [49]; this was supported by the correlation analysis in Table 2, where sucrose to starch ratio displayed a significant positive correlation with nitrogen levels (R^2^ = 0.780; P≤ 0.05). It was also noted that sucrose/starch ratio had a significant positive relationship with protein (R^2^ = 0.709; P ≤ 0.05). These results signify that SPS activity was higher in plants that contained high nitrogen and protein contents. A similar result as in the present study was also observed by Cruz *et al.* [50] on *Ceratina siliqua*.

## 3. Experimental

### 3.1. Experimental Location, Plant Materials and Treatments

The experiment was carried out in growth houses at Field 2, Faculty of Agriculture Glasshouse Complex, Universiti Putra Malaysia (longitude 101° 44’ N and latitude 2° 58’S, 68 m above sea level) with a mean atmospheric pressure of 1.013 kPa. Three-month old *L. pumila* seedlings of var *alata* were left for a month to acclimatize in a nursery until ready for the treatments. Carbon dioxide enrichment treatment plants were exposed to 400 and 1200 µmol^−1^ mol^−1^ CO_2_, started when the seedlings had reached 4 months of age and they were fertilized with three rates of nitrogen, viz. 0, 90 and 270 kg N ha^−1^, applied in the form of urea. The fertilization with nitrogen levels were split into three applications (Table 5). This factorial experiment was arranged in a split plot using a randomized complete block design with CO_2_ levels being the main plot, and nitrogen levels/rates as the sub-plot replicated three times. Each treatment consisted of ten seedlings (Table 5).

**Table 5:** Treatments combination of CO_2_ enrichment and nitrogen fertilization levels of *Labisia pumila* Benth. during the experiment.

CO_2_ levels (µmol mol^−1^)	Nitrogen (kg N Ha^−1^) ^1,2^	Total nitrogen fertilizer per plant ^3^ (g)
400	0	0.00
	90	0.36
	270	1.08
1,200	0	0.00
	90	0.36
	270	1.08

^1^ = Nitrogen source used was urea (46% N); ^2^ = Every nitrogen treatment receives TSP (Triple super phosphate; 46% P) and MOP (muriate of potash; 60% K) at standard rates of 180 kg N ha^−1^; the nitrogen was split into three fertilization phases, and each phase was about 33.3% of total nitrogen fertilizer; ^3^ = Every nitrogen treatment receives TSP (triple super phosphate; 46% P; 0.72 g per plant) and MOP (60% K; 0.51 g per plant) at standard rates of 180 kg N/ha.

### 3.2. Growth House Microclimate and CO_2_ Enrichment Treatment

The seedlings were raised in specially constructed growth houses receiving 12-hour photoperiod and an average photosynthetic photon flux density of 300 µmol m^−2^ s^−1^. Day and night temperatures were recorded at 30 ± 1.0 °C and 20 ± 1.5 °C, respectively, and relative humidity at about 70% to 80%. Vapor pressure deficit ranged from 1.01 to 2.52 kPa. Carbon dioxide at 99.8% purity was supplied from a high–pressure CO_2_ cylinder and injected through a pressure regulator into fully sealed 2 m × 3 m growth houses at 2-hour daily intervals and applied continuously from 0800 to 1000 a.m. [51]. The CO_2_ concentration at different treatments was measured using Air Sense ™ CO_2_ sensors designated to each chamber during CO_2_ exposition period. Plants were watered three to four times a day at 5 min per session to ensure normal growth of plant using drip irrigation with emitter capacity of 2 L h^−1^. The experiment lasted for 15 weeks from the onset of treatment.

### 3.3. Total Phenolics and Total Flavonoids Quantification

The method of extraction and quantification for total phenolics and flavonoids contents followed after Jaafar *et al.* [11]. An amount of ground tissue samples (0.1 g) was extracted with 80% ethanol (10 mL) on an orbital shaker for 120 minutes at 50 °C. The mixture was subsequently filtered (Whatman™ No.1), and the filtrate was used for the quantification of total phenolics and total flavonoids. Folin–Ciocalteu reagent (diluted 10-fold) was used to determine the total phenolics content of the leaf samples. Two hundred µL of the sample extract was mixed with Follin–Ciocalteau reagent (1.5 mL) and allowed to stand at 22 °C for 5 minutes before adding NaNO_3_ solution (1.5 mL, 60 g L^−1^). After two hours at 22 °C, absorbance was measured at 725 nm. The results were expressed as mg g^−1^ gallic acid equivalent (mg GAE g^−1^ dry sample). For total flavonoids determination, a sample (1 mL) was mixed with NaNO_3_ (0.3 mL) in a test tube covered with aluminium foil, and left for 5 minutes. Then 10% AlCl_3_ (0.3 mL)was added followed by addition of 1 M NaOH (2 mL). Later, the absorbance was measured at 510 nm using a spectrophomtometer with rutin as a standard (results expressed as mg g^−1^ rutin dry sample).

### 3.4. Glucose Determination

The anthrone method was used to determine total glucose content in the samples as explained by Hegde and Hofreiter [52]. Dried ground sample (about 0.5 g) was weighed into a 250 mL conical flask and distilled water (10 mL) and 52% perchloric acid (13 mL) were added. The mixture was then shaken using an orbital shaker for 20 minutes. Later, the mixture was transferred into a 100 mL volumetric flask and made up to 100 mL with distilled water. After that, it was filtered into a 250 mL volumetric flask and made up to 100 mL distilled water in another 100 mL volumetric flask. One mL of sample was mixed with anthrone reagent (5 mL) in a test tube. The tube was then placed in a water bath at 100 °C for 12 minutes to obtain a dark green colored solution. The tube was immediately cooled under running tap water and the absorbance was read at 630 nm using a spectrophotometer. Before measurement, a glucose standard was used to produce calibration lines between the spectro-photometer reading and actual glucose content that was expressed as mg g^−1^ glucose dry weight.

### 3.5. Sucrose Determination

Sucrose was measured spectrophotometrically using the method of Edward [53]. Samples (0.5 g) were placed in 15 mL conical tubes, and distilled water added to make up the volume to 10 mL. The mixture was then vortexed and later incubated for 10 minutes. Anthrone reagent was prepared using anthrone (Sigma Aldrich, St Louis, MO, USA, 0.1 g) that was dissolved in 95% sulphuric acid (Fisher Scientific, USA 50 mL). Sucrose was used as a standard stock solution to prepare a standard curve for the quantification of sucrose in the sample. The mixed sample of ground dry sample and distilled water was centrifuged at a speed of 3,400 rpm for 10 minutes and then filtered to get the supernatant. A sample (4 mL) was mixed with anthrone reagent (8 mL) and then placed in a water-bath set at 100 °C for 5 minutes before the sample was measured at an absorbance of 620 nm using a spectrophotometer model UV160U (Shimadzu Scientific, Kyoto, Japan). The soluble sugar in the sample was expressed as mg sucrose g^−1^ dry sample.

### 3.6. Fructose Determination

Fructose was determined by using the method of Ashwel [54]. Plant dry samples (about 0.5 g) were placed in 15 mL conical tubes. Then distilled water (10 mL) was added. The mixture was then shaken for 10 minutes using an orbital shaker. Then the solution (2 mL) was mixed with resorcinol reagent (1 mL) prepared by dissolving resorcinol (1 g) and thiourea (0.25 g) in glacial acetic acid (100 mL). After that, HCl (7 mL) that was prepared by mixing five parts of concentrated HCl with one part of distilled water were mixed with the solution. Then, the tubes were immediately immersed in tap water for 5 minutes before the absorbances were measured at 520 nm. Fructose (Sigma) was used as a standard. The fructose content in the samples was expressed as mg g^−1^ fructose dry weight. 

### 3.7. Starch Determination

Starch content was determined spectrophometrically using a method described by Thayumanavam and Sadasivam [55]. In this method, dry sample (about 0.5 g) was homogenized in hot 80% ethanol to remove the sugar. The sample was then centrifuged at 5,000 rpm for 5 minutes and the residue retained. After that, distilled water (5.0 mL) and 52% perchloric acid (6.5 mL) were added to the residue. Then the solution was centrifuged and the supernatant separated and then filtered with Whatman No. 5 filter paper. The processes were repeated until the supernatant was made up to 100 mL. A sample (100 µL) of the supernatant was added to distilled water until the volume became 1 mL in a test tube. After that, anthrone reagent (4 mL, prepared with 95% sulphuric acid) was added to the test tube. The mixed solution was placed in the water bath at 100 °C for eight minutes and then cooled to room temperature, and then the sample was read at absorbance of 630 nm to determine the sample starch content. Glucose was used as a standard and starch content was expressed as mg glucose equivalent g^−1^ dry sample. 

### 3.8. Total Soluble Sugar and Total Non Structural Carbohydrate (TNC)

The total non structural carbohydrate was calculated as the sum of total soluble sugar and starch content [40].

### 3.9. Total Carbon, Nitrogen and C:N Ratio

Total carbon and C:N ratio were measured by using a CNS 2000 analyzer (Model A Analyst 300, LECO Inc, USA). This was performed by placing 0.05 g of ground leaf sample into the combustion boat. Successively, the combustion boat was transferred to the loader before the sample was burned at 1,350 °C to obtain the reading of total carbon and nitrogen content of the samples.

### 3.10. Phenylalanine-ammonia-lyase (PAL)

Phenylalanine-ammonia-lyase (PAL) activity was measured using the method described by Martinez and Lafuente [56]. The enzyme activity was determined by measuring spectrophotometrically the production of *trans*-cinnamic acid from L-phenylalanine. Enzyme extract (10 µL) was incubated at 40 °C with 12.1 mM L-phenylalanine (90 µL, Sigma) that were prepared in 50 mM Tris-HCl, (pH 8.5). After 15 minutes of reaction, *trans*-cinnamic acid yield was estimated by measuring increase in the absorbance at 290 nm. Standard curve was prepared by using a *trans*-cinnamic acid standard (Sigma) and the PAL activity was expressed as nM *trans*-cinnamic acid µg^−1^ protein hour^−1^.

### 3.11. Protein Determination

Protein content was determined using the method of Bradford [57]. In this method, fresh leaf samples (about 2 g) were cut into pieces using scissors and ground in mortar with 0.05 M Tris buffer (1 mL, pH 8.5) and powdered with liquid nitrogen. The homogenate was then centrifuged at 9,000 rpm for 10 minutes and then stored under refrigeration at 4 °C for 24 hour. After the extraction, supernatant from the samples (about 100 µL) was added to Bradford reagent (3 mL, Sigma, prepared using 10 mL of the reagent diluted with 50 mL distilled water) and then incubated for 5 min before being measured at 595 nm with the spectrophotometer. In this method bovine serum (Sigma) was used as a standard to produce calibration curve between actual protein content and spectrophotometer readings. The protein was expressed as mg g^−1^ protein fresh weight.

### 3.12. Statistical Analysis

Data were analyzed using analysis of variance using SAS version 17. Mean separation test between treatments was performed using Duncan multiple range test and standard error of differences between means was calculated with the assumption that data were normally distributed and equally replicated.

## 4. Conclusions

An increase in secondary metabolites was observed in *Labisia pumila* seedlings under the interaction between high CO_2_ and low nitrogen levels. The results indicate that increasing in total phenolics and flavonoids under high CO_2_ (1,200 µmol mol^−1^) and low nitrogen levels (0 kg N ha^−1^) might be contributed by the increase in erythose-4-phosphate production in the pentose phosphate pathway as elucidated by the increase in fructose content and PAL activities. Simultaneously, the C/N ratio correlated positively with increase in plant secondary metabolites. The sucrose/starch ratio, which is an indication of SPS activity, was, however, found to decrease under low nitrogen levels, probably due to low protein content. 
